# Inference on population history and model checking using DNA sequence and microsatellite data with the software DIYABC (v1.0)

**DOI:** 10.1186/1471-2105-11-401

**Published:** 2010-07-28

**Authors:** Jean-Marie Cornuet, Virgine Ravigné, Arnaud Estoup

**Affiliations:** 1 INRA, UMR CBGP (INRA/IRD/Cirad/Montpellier SupAgro), Campus international de Baillarguet, CS 30016, F-34988 Montferrier-sur-Lez cedex, France; 2 CIRAD, Unité Mixte de Recherche-Biologie et Génétique des Interaction Plante-Parasite, F-34398 Montpellier, France

## Abstract

**Background:**

Approximate Bayesian computation (ABC) is a recent flexible class of Monte-Carlo algorithms increasingly used to make model-based inference on complex evolutionary scenarios that have acted on natural populations. The software DIYABC offers a user-friendly interface allowing non-expert users to consider population histories involving any combination of population divergences, admixtures and population size changes. We here describe and illustrate new developments of this software that mainly include (i) inference from DNA sequence data in addition or separately to microsatellite data, (ii) the possibility to analyze five categories of loci considering balanced or non balanced sex ratios: autosomal diploid, autosomal haploid, X-linked, Y-linked and mitochondrial, and (iii) the possibility to perform model checking computation to assess the "goodness-of-fit" of a model, a feature of ABC analysis that has been so far neglected.

**Results:**

We used controlled simulated data sets generated under evolutionary scenarios involving various divergence and admixture events to evaluate the effect of mixing autosomal microsatellite, mtDNA and/or nuclear autosomal DNA sequence data on inferences. This evaluation included the comparison of competing scenarios and the quantification of their relative support, and the estimation of parameter posterior distributions under a given scenario. We also considered a set of scenarios often compared when making ABC inferences on the routes of introduction of invasive species to illustrate the interest of the new model checking option of DIYABC to assess model misfit.

**Conclusions:**

Our new developments of the integrated software DIYABC should be particularly useful to make inference on complex evolutionary scenarios involving both recent and ancient historical events and using various types of molecular markers in diploid or haploid organisms. They offer a handy way for non-expert users to achieve model checking computation within an ABC framework, hence filling up a gap of ABC analysis. The software DIYABC V1.0 is freely available at http://www1.montpellier.inra.fr/CBGP/diyabc.

## Background

Natural populations are often characterized by complex demographic histories. Their effective sizes and ranges change over time leading to fission and fusion processes that leave signatures on their genetic constitution and structure. One promising prospect of current biology is that molecular data will help us to reveal the complex demographic processes that have acted on populations. The extensive availability of different molecular markers and increased computer power has promoted the development of inferential methods and associated software that have begun to fulfil these expectations [1,2].

Approximate Bayesian computation (ABC) is a recent flexible class of Monte-Carlo algorithms for performing model-based inference [3]. Estimations associated with demographic and genetic models often imply a full likelihood calculation, which is difficult for complex evolutionary scenarios. ABC methods bypass exact likelihood calculations by using summary statistics and massive computer simulations and make it possible to handle large data sets, such as data for hundreds of individuals genotyped at tens of microsatellite loci. The development of ABC has hence generated a sharp increase in the complexity of models used in various fields [4,5]. ABC methods were recently successfully used to make inference on complex models in population and evolutionary biology [6-13], infectious disease epidemiology [14] and system biology [15]. Such inferences mainly include model selection among a finite set of models (evolutionary scenarios) and inferences on the posterior distribution of the parameter of interest under a given model. Whereas several studies have now shown that parameter posterior distributions inferred by ABC are similar to those provided by full-likelihood Bayesian approaches [16-19], the approach is still in its infancy and continues to evolve, and to be improved (reviewed in [4,5,20,21]). In statistical analysis assessing the "goodness-of-fit" of a model (here an evolutionary scenario) with respect to a "real" data set is termed model checking. If a (selected) model has a good fit then the observed data set should look plausible under the posterior predictive distribution of the model [22]. Although useful when doing inferences, model checking is a feature of ABC analyses that has been so far neglected ([5]; but see [23-25]).

Until recently, the ABC approach has remained inaccessible to most biologists because of the complex computations involved. Since 2008, several ABC softwares have been proposed to provide solutions to non-specialist users [26-32]. Cornuet *et al. *[26] developed the software DIYABC in which a user-friendly interface helps non-expert users to perform historical inference using ABC. DIYABC allows considering complex population histories involving any combination of population divergences, admixtures and population size changes, with population samples potentially collected at different times. DIYABC can be used to compare competing evolutionary scenarios and quantify their relative support, and estimate parameters for one or more scenarios. Eventually, it provides a way to evaluate the amount of confidence that can be put into the various estimations. So far, DIYABC applied only to independent autosomal microsatellite data and did not offer users to achieve model checking computation.

This article describes new developments of DIYABC that mainly include (i) the extension of ABC analysis to DNA sequence data in addition or separately to microsatellite data and (ii) the possibility to proceed model checking computation to assess the "goodness-of-fit" of a model within an ABC framework. We used controlled simulated data sets generated under complex evolutionary scenarios to evaluate the interest of mixing autosomal microsatellite, mtDNA and/or nuclear autosomal DNA sequence data. We also used a set of scenarios often considered when making ABC inferences on the routes of introduction of invasive species to illustrate the interest of the model checking option of DIYABC to assess model misfit.

## Methods

### New implementations in DIYABC V1.0

The new version of the software allows the treatment of haploid in addition to diploid data. Five categories of loci (either microsatellites or DNA sequences) can now be analyzed together or separately: autosomal diploid, autosomal haploid, X-linked, Y-linked and mitochondrial. X-linked loci can be used for a haplo-diploid species in which both sexes have been sampled. The data for each type of markers may have been obtained from the same or different individuals. Balanced or non balanced sex ratios can be considered.

Four different mutation models can be chosen for DNA sequence data. For all mutation models, insertion-deletion mutations are not considered mainly because there does not seem to be much consensus on this topic. Concerning substitutions, we have implemented the following models: the Jukes-Cantor [33] one parameter model, the Kimura [34] two parameter model, the Hasegawa-Kishino-Yano [35] and the Tamura-Nei [36] models. The last two models include the ratios of each nucleotide as parameters. However, in order to reduce the number of parameters, these ratios have been fixed to values observed in the data for each DNA sequence locus. Consequently, this leaves two and three variable parameters for the Hasegawa-Kishino-Yano (HKY) and Tamura-Nei (TN), respectively. Summary statistics can be chosen for DNA sequence data among a set of 14 statistics detailed in the notice document available at http://www1.montpellier.inra.fr/CBGP/diyabc. As for microsatellite loci, DNA sequence summary statistics are averaged for a type of sequence loci (e.g. nuclear DNA sequence loci). This allows reducing the total number of summary statistics as the latter may quickly increase when considering summary statistics independently for each sequence locus.

With respect to microsatellite loci, the possibility of uneven insertion/deletion events (i.e. allele lengths are sometimes not multiple of the motif length implying that there has been single nucleotide insertion-deletion mutations in the flanking regions of microsatellites [37]) is now better taken into account in inferences as this type of mutation events is not considered anymore as a nuisance parameter but can be estimated by considering a mean mutation rate (mean *μ*SNI) drawn from various prior distributions and some individual locus mutation rates drawn from some Gamma distribution with mean = mean *μ*SNI.

A new option called "evaluate scenario-prior combination" allows checking whether some of the models together with the chosen prior distributions have the potential to generate a subset of summary statistics close to the observed summary statistics (i.e. the target statistics obtained from the data set on which one wants to make inferences). In the first analysis proposed by this option, a principal component analysis is performed in the space of summary statistics on at most 10,000 simulated data sets and the target (observed) data set is added on each plane of the analysis in order to evaluate how the latter is surrounded by the simulated data sets. In addition to this global approach, there is a second one in which each summary statistic of the observed data set is ranked against those of the simulated data set. This second analysis helps finding which aspects of the model (including prior) is problematic. For instance, a grossly underestimated genetic distance (in simulated data sets compared to the observed one) may suggest a misspecification of the prior distribution of a divergence time between two populations or of the mean mutation rate of the markers. To our experience, using this new option before running a full ABC treatment with DIYABC is a convenient and easy way to reveal noticeable misspecification of prior distributions and/or models (see Additional file 1 for an illustration).

Following Gelman *et al*. ([22] pp 159-163), we implemented a new option in DIYABC V1.0, called "model checking", to measure the discrepancy between a combination of a model and parameter posterior distributions and a "real" data set by considering various sets of test quantities. These test quantities can be chosen among the large set of ABC summary statistics proposed in DIYABC V1.0. Details regarding these new computations are given below in the methods and results sections entitled *Model checking*.

DIYABC V1.0 was written in Delphi 2009 and runs under a 32-bit Windows operating system. It is worth stressing that this new version of the software was recoded in order to use a multithread technology allowing the exploitation of multicore/multiprocessor computers. This is especially useful when building the reference table and for several other intensive computation steps, such as the multinomial logistic regression. Such improvements allow a substantial gain of speed for ABC treatments when using multicore/multiprocessor computers, which now are found in most biology research laboratories.

### Mixing microsatellite, mtDNA and/or nuclear DNA sequence data

**Figure 1:**
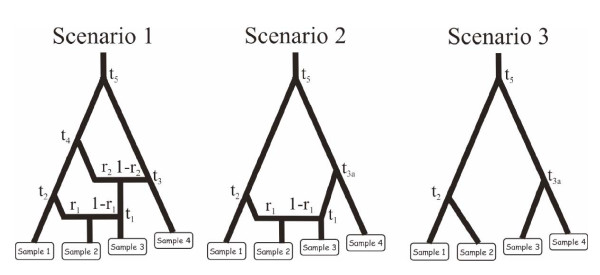
**Evolutionary scenarios to evaluate the interest of mixing microsatellite with mtDNA and/or nuclear DNA sequence data**. The three presented scenarios involve different number of divergence and admixture events that occurred at recent to ancient times. Scenario 1 includes six populations, the four that have been sampled (30 diploïd individuals per population) and two unsampled parental populations in the admixture events. The two admixed populations are those represented by samples 2 and 3. Scenario 2 and 3 include five and four populations, respectively. Scenario 2 includes a single admixed population represented by sample 2. Scenario 3 does not include any admixed population. For all scenarios, samples 3 and 4 have been collected 2 and 4 generations earlier than the first two samples, hence their slightly upward locations on the graphs. Time is not at scale. See text of Methods (section "Mixing microsatellite, mtDNA, and/or nuclear DNA sequence data") for details regarding prior distributions of microsatellite and sequence markers.

In order to evaluate the interest of mixing microsatellite loci with mtDNA and/or nuclear DNA sequence data, we used simulated data sets generated under three complex evolutionary scenarios similar to those presented in Cornuet *et al. *[26]. These scenarios involved different number of divergence and admixture events that occurred at recent to ancient times (see Figure 1). We evaluated the potential of different types of data sets (ten autosomal microsatellite loci, one mtDNA sequence of 1,000 nucleotides, five nuclear autosomal DNA sequences of 1,000 nucleotides each, and all combinations of two and three types of markers) to compare the three competing scenarios and estimate parameters under each scenario.

Prior distributions of demographic parameters were as followed: Uniform[10; 10000] for effective population sizes (similar for all populations), Uniform[1; 100] for *t1*, Uniform[100; 1000] for *t2*, Uniform[5000; 50000] for *t3*, *t3a*, and *t4 *(with *t4 *>*t3*), Uniform[50000; 500000] for *t5*, and Uniform[0.1; 0.9] for *r1 *and *r2*. For microsatellite markers, the ten loci were assumed to follow a generalized stepwise mutation model (GSM [37]) with two parameters: the mean mutation rate (mean *μ*) and the mean parameter of the geometric distribution of the length in number of repeats of mutation events (mean *P*) drawn from Uniform[10-4; 10-3] and Uniform[0.1; 0.3] prior distributions, respectively. Each locus has a possible range of 40 contiguous allelic states and was characterized by individual *μ_loc _*and *P_loc _*values drawn from Gamma(mean = mean *μ *and shape = 2) and Gamma(mean = mean *P *and shape = 2) distributions, respectively [12]. For DNA sequence loci (one mtDNA locus and five nuclear DNA loci), the sequences were assumed to follow the two parameter model of Kimura [34] with a fraction of constant sites (those that cannot mutate) fixed to 10% and the shape parameter of the Gamma distribution of mutations among sites equal to 2. For each sequence locus (1,000 nucleotide per sequence), the mean mutation rate per nucleotide and generation was drawn in a Uniform[10^-8^; 10^-7^] and a Uniform[10^-9^; 10^-8^] for the mtDNA and nuclear sequences, respectively [53].

The summary statistics for microsatellite loci were the mean number of alleles, expected heterozygosity [38] and allele size variance per population, *F*_ST _values and genetic distance (*δμ*)^2 ^between pairs of populations [39,40] and the maximum likelihood estimate of admixture proportion [41]. The summary statistics for DNA sequence loci were (i) the number of distinct haplotypes, the number of segregating sites, the mean pairwise difference, the variance of the number of pairwise differences (all statistics computed within each sample), (ii) the number of distinct haplotypes, the number of segregating sites (all statistics computed in samples pooled by pair), and (iii) the *F*_ST _between pairwise samples (computed as in [42]) and an adaptation for sequence data of the maximum likelihood estimate of admixture proportion of Choisy *et al. *[41]. Mean values of such statistics were computed over loci grouped by category (microsatellites, nuclear DNA sequences and mitochondrial DNA sequence).

For each combination of marker type, we simulated 10^6 ^data sets for each of the three competing scenarios. For each competing scenario, we simulated 500 test data sets (i.e. pseudo-observed data sets) drawing demographic and marker parameter values in the same distributions as those used to generate the reference table (see legend of Figure 1). For model comparison, we estimated the posterior probabilities of the competing scenarios using a polychotomous logistic regression on the 1% of simulated data sets closest to the observed data set [26]. Posterior probabilities of the three scenarios were used to compute type I and II errors in the choice of each scenario. For instance, let us consider the estimation of type I and type II errors when choosing scenario 2 as the true scenario. To do so, we simulate 500 data sets according to scenario 1, 2 and 3. Then we count the proportion of times that scenario 2 has not the highest posterior probability among the three competing scenarios when it is the true scenario (type I error, estimated from test data sets simulated under scenario 2) or the proportion of times that scenario 2 has highest posterior probability when it not the true scenario (type II error, estimated from test data sets simulated under scenarios 1 and 3).

We then estimated the posterior distributions of parameters under the most complex scenario (i.e. scenario 1) using a local linear regression on the 1% closest simulated data sets and applying a *logit *transformation to parameter values [3,26]. We evaluated the precision of parameter estimation by computing the median of the absolute error divided by the true parameter value of the 500 pseudo-observed data sets simulated under scenario 1 using the median of the posterior distribution as point estimate (RMAE). All computations were processed using DIYABC V1.0.

### Model checking

A combination of a model and parameter posterior distributions is acceptable only if the observed data look similar to replicated data generated under this model-posterior combination (i.e. under the posterior predictive distribution; [5,20]). To put it another way, the observed data should look plausible under the posterior predictive distribution. This is really a self-consistency check: an observed discrepancy can be due to model misfit (demographic and/or marker models) or chance. Following Gelman *et al*. ([22] pp 159-163), we implemented an option in DIYABC V1.0 to evaluate the discrepancy between a model-posterior combination and a target (observed) data set by considering various sets of test quantities. These test quantities are chosen among the set of ABC summary statistics proposed in DIYABC V1.0 (see the notice document available at http://www1.montpellier.inra.fr/CBGP/diyabc for an illustration). For each test quantities (*t *corresponding to the chosen summary statistics), a lack of fit of the observed data with respect to the posterior predictive distribution can be measured by the cumulative distribution function values of each test quantities defined as Prob(*t*_simulated _<*t*_observed_). Tail-area probability, or *p*-value, can be easily computed for each test quantities as Prob(*t*_simulated _<*t*_observed_) and 1.0 - Prob (*t*_simulated _<*t*_observed_) for Prob (*t*_simulated _<*t*_observed_) ≤ 0.5 and > 0.5, respectively [22]. Such *p-*values represent the probability that the replicated data (simulated ABC summary statistics) could be more extreme than the observed data (observed ABC summary statistics). Too many observed summary statistics on the tails of distributions would cast serious doubts on the adequacy of the model-posterior combination. Because *p*-values are computed for a number of test statistics, we used the method of Benjamini and Hochberg [43] to control the false discovery rate (see [44] for a comparative study of several methods dealing with false discovery rate control and [23] for an application in the context of an ABC study). An alternative way to combine *p*-values across test statistics has been recently proposed [25].

One complication with inferences using ABC is that at least some and sometimes all summary statistics used as tests quantities have already been used during the inference steps (model discrimination and estimation of parameters). There is a risk of over-estimating the quality of the fit by using the same statistics twice. This problem which clearly arises within an ABC framework is actually a general one in statistical inference. As underlined in many text books in statistics (e.g. [22,45] and see [5]), it is advised against performing model checking using information that have already been used for training (i.e. model fitting). Optimally, model checking should be based on test quantities that do not correspond to the summary statistics that have been used for previous inferential steps; this is naturally possible with DIYABC as the package propose a large choice of summary statistics. The choice of the two sets of statistics remains a difficult issue that still needs to be thoroughly investigated (and that we will not investigate here). In practice, one could advise users to choose the set of statistics for the model discrimination and parameter estimation step and the set of statistics for the model checking step before they embark on the first step. Moreover, it seems sensible that both sets include statistics describing genetic variation both within and between populations.

**Figure 2:**
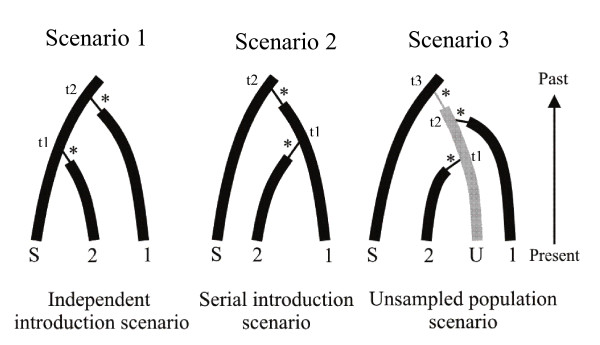
**Evolutionary scenarios to illustrate model checking**. The three presented scenarios are often compared when making ABC inferences on the routes of introduction of invasive species. S is the source population in the native area, and U, the unsampled population in the introduced area that is the source of populations 1 and 2 in scenario 3. The stars indicate the bottleneck events occurring in the first few generations following introductions. We here considered that the dates of first observation were well known so that divergence times could be fixed at 5, 10, 15 and 20 generations for *t1*, *t2*, *t3 *and *t4*, respectively. The data sets consisted of simulated genotypes at 20 (independent) microsatellite loci obtained from a sample of diploid individuals collected from the invasive and source populations (30 individuals per population). The pseudo-observed test data set that we analyzed to illustrate model checking was simulated under scenario 3 with an effective population size (*NS*) of 10,000 diploid individuals in all populations except during the bottleneck events corresponding to an effective population size (*NFi*) of 10 diploid individuals for 5 generations. Prior distributions for ABC analyses (discrimination of scenarios and estimation of posterior distribution of parameters) were as followed: Uniform[1000; 20000] for and logUniform[2; 100] for the demographic parameters *NS *and *NFi*, respectively, and same distributions as those given in the text of Methods (section "Mixing microsatellite, mtDNA, and/or nuclear DNA sequence data") for microsatellite markers.

To illustrate this new model checking option of DIYABC V1.0, we have chosen a set of basic scenarios considered when making ABC inferences on the routes of introduction of invasive species [46,47]. We considered three models in which two invasive populations originate from the same source population. These populations may be related through three different scenarios: the independent introduction scenario, the serial introduction scenario and the unsampled population scenario (Figure 2). In the independent or serial introduction scenarios, all the populations concerned were sampled, but in the unsampled population scenario, the two invasive populations were founded independently from an undetected and hence unsampled population, itself introduced from the source. It is worth stressing that although previous studies have shown that some invasive populations may remain undetected but may play important role in the invasion dynamics of some species [48,49], the unsampled population scenario is often not considered. If only the traditional independent and serial introduction scenarios are compared, a "real" data set obtained under the unsampled population scenario will erroneously fit one of the two competing scenario with often a high posterior probability (see results section and [47]). Here we used a single, randomly chosen, pseudo-observed test data set simulated under the unsampled population scenario to illustrate the interest of the model checking option of DIYABC.

Standard ABC analyses (estimation of model probabilities and of parameter posterior distributions) were first performed on the above test data set as described previously (i.e. in the section "Mixing microsatellite, mtDNA and/or nuclear DNA sequence data"). We drew parameter values from the prior distributions described in the legends of Figure 2 and used the summary statistics described in Table 1. Model checking computations were then processed by simulating 10,000 data sets under each studied model-posterior combination, with sets of parameter values drawn with replacement among the 10,000 sets of the posterior sample. We computed two groups of test quantities: a first group of summary statistics already used for model discrimination and estimation of parameter posteriors and a second group of summary statistics not previously used for inferences. Each observed summary statistics was then ranked and given cumulative distribution function values among the corresponding sample of summary statistics obtained through the above simulation, providing an estimation of *p*-value for each summary statistics. In addition, a principal component analysis (PCA) was performed in the space of summary statistics. Principal components were computed considering 10,000 data sets simulated with parameter values draw from the prior. Then the target (observed) data set as well as the 1,000 data sets simulated from the posterior distributions of parameters were added to each plane of the PCA. If the model-posterior combination fits well the observed data set, one should see on each PCA plane a wide cloud of data sets simulated from the prior, with the observed data set in the middle of a small cluster of data sets generated from the posterior predictive distribution. All computations and illustrations were processed using DIYABC V1.0.

**Table 1:** Model checking for introduction scenarios 1, 2 and 3.

			**Probability (*t***_**simulated**_**<*t***_**observed**_**)**		
			
	Test quantity (*t*)	Observed value	Scenario 1	Scenario 2	Scenario 3
Test quantities	NAL_S	13.6000	0.7275	0.2871	0.6235
corresponding	NAL_1	3.4000	0.7542	0.9865 (*)	0.4252
to thesummary	NAL_2	3.6500	0.6455	0.4102	0.4761
statistics used	HET_S	0.8429	0.5621	0.2471	0.4488
to discriminate	HET_1	0.5151	0.4938	0.9890 (*)	0.4339
among	HET_2	0.5725	0.9125	0.9188	0.8221
scenarios and	MGW_S	0.8242	0.3593	0.7656	0.5230
compute	MGW_1	0.4072	0.3782	0.6713	0.4524
parameter	MGW_2	0.4834	0.6117	0.8499	0.7297
posterior	FST_S_1	0.2170	0.7882	0.0371 (*)	0.8105
distributions	FST_S_2	0.2050	0.6180	0.4606	0.6052
	FST_2_3	0.1761	0.0001 (***)	0.9580 (*)	0.6289

Test quantities	VAR_S	21.7561	0.7476	0.2538	0.6209
corresponding	VAR_1	9.3385	0.4861	0.3561	0.3598
to summary	VAR_2	9.5277	0.5232	0.1792	0.3748
statistics NOT	LIK_1_S	38.5648	0.7867	0.4503	0.7240
used to	LIK_1_2	31.7504	0.0001 (***)	1.0000 (***)	0.7162
discriminate	LIK_2_1	32.1075	0.0001 (***)	0.9850 (*)	0.7836
among	H2P_S_1	0.7734	0.6563	0.8411	0.6115
scenarios and	H2P_S_2	0.7993	0.9231	0.8239	0.8664
compute	H2P_1_2	0.6020	0.0315 (*)	0.9975 (**)	0.7193
parameter	DAS_S_1	0.1329	0.2298	0.4582	0.2639
posterior	DAS_S_2	0.1099	0.0559	0.1681	0.0816
distributions	DAS_1_2	0.3402	1.0000 (***)	0.0001 (***)	0.2529

**Figure 3:**
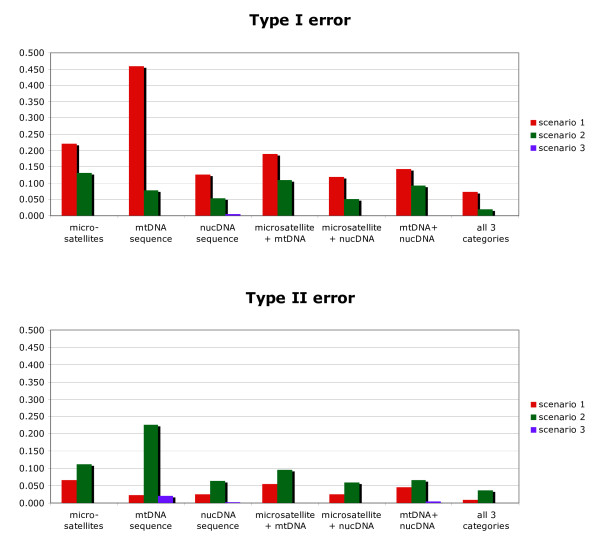
**Confidence in discriminating evolutionary scenarios using microsatellite, mtDNA and/or nuclear DNA sequence data**. The three compared scenarios are detailed in Figure 1. Type I error: exclude scenario x when it is actually scenario x. Type II error: choose scenario x when it is not scenario x. Results are based on 500 simulated data sets per scenario with parameter values drawn from the same distributions as the prior distributions given in the legend of Figure 1.

Evolutionary scenarios 1, 2 and 3 are detailed in Figure 3. The single "pseudo-observed" test data set analyzed here was simulated under scenario 3. The probability (*t*_simulated _<*t*_observed_) given for each test quantities (*t*) was computed from 10,000 data sets simulated from the posterior distributions of parameters obtained under a given scenario. Corresponding tail-area probabilities, or *p*-values, of the test quantities (*t*) can be easily obtained as Prob(*t*_simulated _<*t*_observed_) and 1.0 - Prob (*t*_simulated _<*t*_observed_) for Prob (*t*_simulated _<*t*_observed_) ≤ 0.5 and > 0.5, respectively [22]. The test quantities correspond to the summary statistics used to discriminate among scenarios and compute the posterior distributions of parameters or to other statistics. NAL_*i *= mean number of alleles in population *i*, HET_*i *= mean expected heterozygosity in population *i *[38], MGW_*i *= mean ratio of the number of alleles over the range of allele sizes [54], FST_*i*_*j *= *F*_ST _value between populations *i *and *j *[39], VAR_*i *= mean allelic size variance in population *i*, LIK_*i*_*j *= mean individual assignment likelihoods of population *i *assigned to population *j *[22], H2P_*i*_*j *= mean expected heterozygosity pooling samples from populations *i *and *j*, DAS_*i*_*j *= shared allele distance between populations *i *and *j *[55]. Populations *i *and *j *correspond to populations S, 1 or 2 in Figure 3. *, **, *** = tail-area probability < 0.05, < 0.01 and < 0.001, respectively. Significant tail-area probabilities after applying the false discovery rate correction method of Benjamini and Hochberg [43] are given in bold italic characters.

## Results and Discussion

### Mixing microsatellite, mtDNA and/or nuclear DNA sequence data

Results dealing with the discrimination among a finite set of competing complex scenarios are summarized in Figure 3. When considering the confidence in scenario choice for each type of markers taken separately, we found that the lowest error rates were obtained for different types of markers depending on the type of error and scenario considered. The lowest type I error rates were obtained with the nuclear sequences for scenarios 1 and 2, and microsatellites or mtDNA for scenario 3. The lowest type II error rates were obtained with mtDNA for scenario 1, the nuclear sequences for scenario 2 and microsatellites for scenario 3. Some differences in error rates between markers were small, however, and hence not significant using Fisher exact tests (e.g. type II errors for scenario 1 were equal to 0.023 and 0.024 for mtDNA and nuclear sequences, respectively). MtDNA displayed contrasted error rates depending on the scenario considered, with sometimes large error values; for instance a type I error of 0.458 for scenario 1 and a type II of error 0.225 for scenario 2. These large error rates were probably due to the fact that mtDNA data correspond to a single locus and hence to a single gene genealogy subject to substantial stochastic variation [50]. Adding sequence data (mtDNA or nuclear DNA) to microsatellite data globally decreased type I errors (especially for scenario 2 for which type I error was two times lower) and type II errors (especially for scenario 1 for which type I error was two times lower). For all three scenarios, the lowest type I and II error values were obtained when combining the three types of markers.

**Figure 4:**
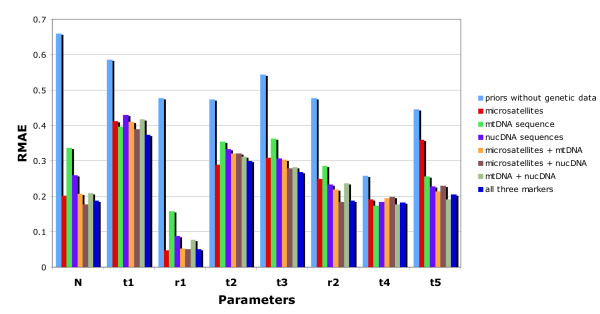
**Precision in parameter estimation using microsatellite, mtDNA and/or nuclear DNA sequence data under scenario 1**. Results are based on 500 pseudo-observed test data sets simulated and estimated under scenario 1 presented in Figure 1, with parameter values drawn from the same distributions as the prior distributions given in the legend of Figure 1. The demographic parameters *N*, *t1*, *t2*, *t3*, *t4*, *t5*, *r1 *and *r2 *are detailed in Figure 1. RMAE: relative median absolute errors. The blue columns correspond to the "base-level" RMAE values obtained using only the prior information on parameters (no genetic data).

Results dealing with the estimation of parameters under scenario 1 are summarized in Figure 4. Whatever the type and combination of markers, the molecular data provided substantial information for all parameters except the divergence times t1, t2 and t4 for which the level of information remained low. For the latter parameters the relative median absolute errors (RMAE) were only slightly lower than those computed as base level using only the prior information on parameters (blue bars in Figure 4). This is not surprising since t1 corresponds to a very recent time of admixture (< 100 generations) and t2 or t3 correspond to divergence times for which one of the two diverging populations has not been sampled.

We found that, depending on the parameter considered, cumulating the information provided by different markers translated into a decrease, an increase or, most frequently, an absence of noticeable variation of the RMAE values compared to that obtained with the most informative genetic marker (Figure 4 and see Additional file 2 for an illustration of the variation of RMAE values expected by chance between different replicates of 500 pseudo-observed data sets). Although each category of markers is different and genealogically independent, the genetic variation at these markers is constrained by the fact that they share the same evolutionary history (i.e. same historical and demographic parameters) so that information provided by each category is not expected to sum up. For all demographic parameters, the lowest RMAE values were obtained, however, when combining the three categories of markers; but in many cases, one or the other category (depending on the considered parameter), taken alone, provided almost the same precision.

We found that adding sequence data substantially improved the quality of the estimations of some parameters in comparison to results obtained with microsatellites only. This was particularly true for the most ancient divergence time *t5 *for which RMAE values decreased by 41%, 36% and 47% when adding a single mtDNA sequence, five nuclear sequence and both types of sequences, respectively. Only small decreases of RMAE values were observed for moderately ancient events such as the divergence time *t3 *and the admixture rate *r2*. This result underlines the interest of using low mutating and seldom homoplasious sequence data for making inferences on ancient historical events. In agreement with this, the two RMAE values for *t5 *obtained with the mtDNA data sets and the nuclear sequence data sets were lower than that obtained for microsatellite loci only. Due to their mutation modalities (high mutation rates with allele size homoplasy and constraints [37]), it is not surprising that microsatellite loci performed poorly for ancient evolutionary events [51,52]. On the opposite, microsatellite markers provided substantially better estimation than mtDNA or nuclear sequence for the most recent admixture rate (*r1*). The RMAE values for the mtDNA sequence and the nuclear sequences were two to three times larger than those obtained with microsatellite only for this parameter. As a result, the addition of mtDNA or nuclear sequences to microsatellite data did not bring any progress in terms of RMAE for *r1*. This result holds to a lesser extent for the effective population size *N*.

### Model checking

When considering altogether the three scenarios in our model discrimination analysis, we found that our (single) pseudo-observed test data set generated under the unsampled population scenario (scenario 3 in Figure 2) was unambiguously assigned to the correct scenario with a high posterior probability (p = 0.9967, 95% CI [0.9958, 0.9976]). When only scenarios 1 and 2 were proposed for posterior probability estimation then the same test data set generated under scenario 3 was assigned to the incorrect scenario 2 with a high posterior probability (p = 0.9999, 95% CI [0.9998, 1.0000]). Additional ABC treatments achieved on larger sets of pseudo-observed test data sets (*n *= 1,000) confirmed that if only the traditional independent and serial introduction scenarios are considered, a data set obtained under the unsampled population scenario will erroneously be chosen, with often a high posterior probability to one of the two competing scenario; scenario 2 is chosen for 55% and 63% of the data sets generated under scenario 3 when simulating test data sets using the same fixed parameter values than the above single test data set and when drawing parameter values in the same distribution than those chosen as priors, respectively.

Focusing on our single pseudo-observed test data set simulated under scenario 3, we evaluated in details the interest of the model checking option of DIYABC V1.0 to assess model misfit. We found that none of the twelve test quantities had low tail probability values when applying the model checking option to the (true) scenario 3 (last column of Table 1). In contrast, one to several test quantities had low tail-area probabilities (sometimes lower than p = 0.001) when applying the model checking option to (incorrect) scenarios 1 or 2, hence casting serious doubts on the adequacy of the tested model-posterior combination. We found some indication of a risk of over-estimating the quality of the fit by using as test quantities the same summary statistics already used during the inference steps (model discrimination and posterior estimation of parameters); see Table 1. The proportion of test quantities with low tail-area probabilities was indeed larger when using summary statistics not previously used for inference. A close examination of which summary statistics displayed low tail-area probabilities provides some insights on which aspects of the models 1 and 2 are problematic. In the studied case, outlying statistics correspond to an overestimated genetic differentiation in simulated data sets compared to the observed one between the introduced populations 1 and 2 for scenario 1, whereas it correspond to an underestimated genetic differentiation for scenario 2. This pattern is in agreement with the specificities of the "true" scenario 3 (partial genealogical dependency between populations 2 and 3 through the unsampled population) relatively to scenario 1 (weak genealogical dependency between the independently introduced populations 2 and 3) and scenario 2 (strong genealogical dependency between the serially introduced populations 2 and 3); see Figure 3.

We further inspected the fit/misfit of models by performing several principal component analysis on the test quantities obtained with the different model -posterior combinations together with the pseudo-observed test data set simulated under the unsampled population scenario (Additional file 3). In agreement with the quantitative results summarized in Table 1, the PCA points of the test quantities obtained from the model-posterior combination corresponding to the (true) scenario 3 were nicely grouped and centred on the target point corresponding to the pseudo-observed test data set. This configuration holds when considering either previously used or unused ABC summary statistics as test quantities. When considering scenarios 1 and 2, we found that the target point of the "pseudo-observed" test data set was positioned at best on the border of the cloud of PCA points of the test quantities corresponding to the summary statistics previously used for ABC analyses. Interestingly enough, the target point was clearly outside the cluster when considering unused summary statistics as test quantities.

The model checking analysis of other pseudo-observed test data sets provided results (quantitatively) similar to those presented in the Table 1 and Additional file 3 (results not shown).

## Conclusions

The software DIYABC V1.0 offers a user-friendly interface allowing non-expert users to perform additional and more accurate inferences using ABC than its previous version. The new implementations allow the treatment of haploid in addition to diploid data and allow making inferences from DNA sequence data (without recombination) in addition or separately to microsatellite data. The possibility of mixing different types of molecular markers (including autosomal, X or Y-linked loci, and mtDNA loci) should prove useful when considering complex evolutionary scenarios involving both recent and ancient historical events. Finally, DIYABC V1.0 offers non-specialist users a handy way to achieve model checking computation (i.e. the assessment of the "goodness-of-fit" of a model - posterior combination with respect to a target data set), a feature of ABC analysis that has been so far neglected. These new software developments significantly enlarge the tool box available to biologists to make ABC inferences on more complex and hence more realistic demographic processes that have acted on natural populations. The main limitations of the current version of DIYABC are the assumed absence of migration among populations after they have diverged, the impossibility to consider other reproduction systems than standard sexuality as well as evolutionary neutrality of markers. Next developments will aim at progressively removing these limitations.

## Authors' contributions

JMC and AE carried out the analyses, wrote the paper and jointly developed the software DIYABC v1.0 with VR. All authors read and approved the final manuscript.

## Supplementary Material

Additional file 1 **Pre-evaluation of model-prior combinations: two examples**. **Pre-evaluation of model-prior combinations: example 1**. A single test pseudo-observed data set (10 microsatellite loci) was first simulated under a model of a single population (sample size of 30 diploid individuals) with effective size *N *= 10,000. Microsatellite loci were assumed to follow a generalized stepwise mutation model (GSM [37]) with a mean mutation rate (mean *μ*) equal to 5 × 10^-4 ^and a mean parameter of the geometric distribution of the length in number of repeats of mutation events (mean *P*) equal to 0.22. Each locus was given a possible range of 40 contiguous allelic states and was characterized by individual *μ_loc _*and *P_loc _*values drawn from Gamma(mean = mean *μ *and shape = 2) and Gamma(mean = mean *P *and shape = 2) distributions, respectively [12]. For ABC analysis of the test data set, we used the same population and marker models, and prior distributions of demographic parameters were as followed: Uniform[10; 1000] (figure A) or Uniform[2000; 20000] (figure B) for *N*, Uniform[10^-4^; 10^-3^] and Uniform[0.1; 0.3] for mean *μ *and mean *P*, respectively. We choose three summary statistics (*s*): mean number of alleles, mean expected heterozygosity [38] and mean allele size variance per population. PCA on summary statistics (A and B) and probability (*s*_simulated _<*s*_observed_) for each summary statistics (C) were computed from 10,000 simulations, randomly drawing parameter values from priors. **Pre-evaluation of model-prior combinations: example 2**. A single pseudo-observed test data set (10 microsatellite loci) was first simulated under a model of two populations (sample size of 30 diploid individuals per population) splitting at time *t *= 10,000 generations from an ancestral population, without subsequent migration. For all populations the effective size was *N *= 1,000. For ABC analysis of the test data set, we used the same population and marker models, and prior distributions of demographic parameters were as followed: Uniform[100; 1000] (figure D) or Uniform[2000; 20000] (figure E) for *t*, and Uniform[100; 2000] for *N*. The mutation model and priors for microsatellite markers are the same as in example 1. We choose eight summary statistics (*s*): mean number of alleles, mean expected heterozygosity [38] and mean allele size variance of each population sample, and *F*_ST _values and genetic distances (*δμ*)^2 ^between pairs of populations [39,40]. PCA on summary statistics (D and E) and probability (*s*_simulated _<*s*_observed_) for each of the summary statistics (F) were computed from 10,000 simulaxtions, randomly drawing parameter values from priors. Click here for file

Additional file 2 **Evaluation of the variation of RMAE values expected by chance between different replicates of 500 pseudo-observed data sets**. relative median absolute errors (RMAE) were computed for 10 replicates of 500 pseudo-observed data sets simulated under scenario 1. The data sets include 20 (independent) microsatellite loci and were generated under scenario 1 presented in Figure 1. Parameter values were drawn from the same distributions than the prior distributions given in the legend of Figure 1. The demographic parameters *N*, *t1, t2, t3, t4, t5, r1 *and *r2 *are detailed in Figure 1. Standard deviation of RMAE values were equal to 0.009, 0.019, 0.004, 0.017, 0.012, 0.013 and 0.014 for *N*, *t1, t2, t3, t4, t5, r1 *and *r2*, respectively. Similar levels of RMAE variation among replicates of 500 pseudo-observed data sets were obtained for other categories of genetic markers (mtDNA and nuclear sequences) and combinations of categories of markers (results not shown). Click here for file

Additional file 3 **Principal component analysis of test quantities when processing model checking for the introduction scenarios 1, 2 and 3**. The scenarios 1, 2 and 3 are detailed in Figure 2. The pseudo-observed test data set analyzed here was simulated under scenario 3. PCA were processed on the test quantities corresponding to the summary statistics used to discriminate among scenarios and compute the posterior distributions of parameters (a) or on other statistics (b). The summary statistics used as test quantities are detailed in the legend of Table 1. Click here for file
