# Supplementary material for: Inference on population history and model checking using DNA sequence and microsatellite data with the software DIYABC (v1.0)
Source: BMC Bioinformatics. 2010 Jul 28;11:401. doi: 10.1186/1471-2105-11-401 (PMC2919520; doi:10.1186/1471-2105-11-401)
Supplement: Additional file 1 — Pre-evaluation of model-prior combinations: two examples. Pre-evaluation of model-prior combinations: example 1. A single test pseudo-observed data set (10 microsatellite loci) was first simulated under a model of a single population (sample size of 30 diploid individuals) with effective size N = 10,000. Microsatellite loci were assumed to follow a generalized stepwise mutation model (GSM [37]) with a mean mutation rate (mean μ) equal to 5 × 10^-4^ and a mean parameter of the geometric distribution of the length in number of repeats of mutation events (mean P) equal to 0.22. Each locus was given a possible range of 40 contiguous allelic states and was characterized by individual μloc and Ploc values drawn from Gamma(mean = mean μ and shape = 2) and Gamma(mean = mean P and shape = 2) distributions, respectively [12]. For ABC analysis of the test data set, we used the same population and marker models, and prior distributions of demographic parameters were as followed: Uniform[10; 1000] (figure A) or Uniform[2000; 20000] (figure B) for N, Uniform[10-4; 10-3] and Uniform[0.1; 0.3] for mean μ and mean P, respectively. We choose three summary statistics (s): mean number of alleles, mean expected heterozygosity [38] and mean allele size variance per population. PCA on summary statistics (A and B) and probability (ssimulated <sobserved) for each summary statistics (C) were computed from 10,000 simulations, randomly drawing parameter values from priors. Pre-evaluation of model-prior combinations: example 2. A single pseudo-observed test data set (10 microsatellite loci) was first simulated under a model of two populations (sample size of 30 diploid individuals per population) splitting at time t = 10,000 generations from an ancestral population, without subsequent migration. For all populations the effective size was N = 1,000. For ABC analysis of the test data set, we used the same population and marker models, and prior distributions of demographic parameters were as follow [file 1471-2105-11-401-S1.PDF]

Cornuet et al BMC Bioinformatics – supplementary data file 1

Pre-evaluation of model-prior combinations: example 1. A single pseudo-observed test data set (10 microsatellite loci) was first simulated under a model of a single population (sample size of 30 diploid individuals) with effective size N = 10,000. The microsatellite loci followed a generalized stepwise mutation model (GSM; [1]) with a mean mutation rate (mean μ) equal to 5x10^{-4} and the mean parameter of the geometric distribution of the length in number of repeats of mutation events (mean P) equal to 0.22. Each locus has a possible range of 40 contiguous allelic states and was characterized by individual μ_{loc} and P_{loc} values drawn from Gamma(mean=mean μ and shape=2) and Gamma(mean=mean P and shape=2) distributions, respectively [2]. For ABC analysis of the test data set, we used the same population and marker models, and prior distributions of demographic parameters were as followed: Uniform[10; 1000] (figure A) or Uniform[2000; 20000] (figure B) for N, Uniform[10^{-4}; 10^{-3}] and Uniform[0.1; 0.3] for mean μ and mean P, respectively. We choose three summary statistics (s): the mean number of alleles, expected heterozygosity [3] and allele size variance per population. PCA on summary statistics (A and B) and probability ($s_{\text{simulated}}$<s_{observed}) for each of the summary statistics (C) were computed from 10,000 simulations, randomly drawing parameter values into priors.

(A) "True" value of N = 10,000 – "incorrect" prior on N (Uniform[10; 1000]): note that the observed data set (large yellow dot) is positioned outside the cloud of simulated data sets (small dots)

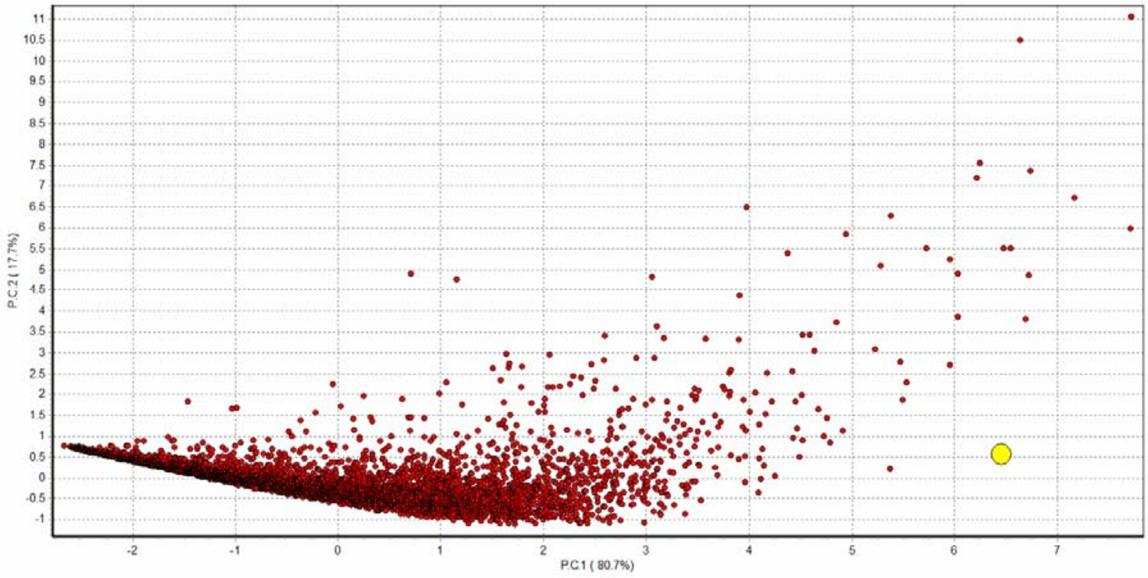

(B) "True" value of N = 10,000 – "correct" prior on N (Uniform[2000; 20000]): note that the observed data set (large yellow dot) is positioned within the cloud of simulated data sets (small dots)

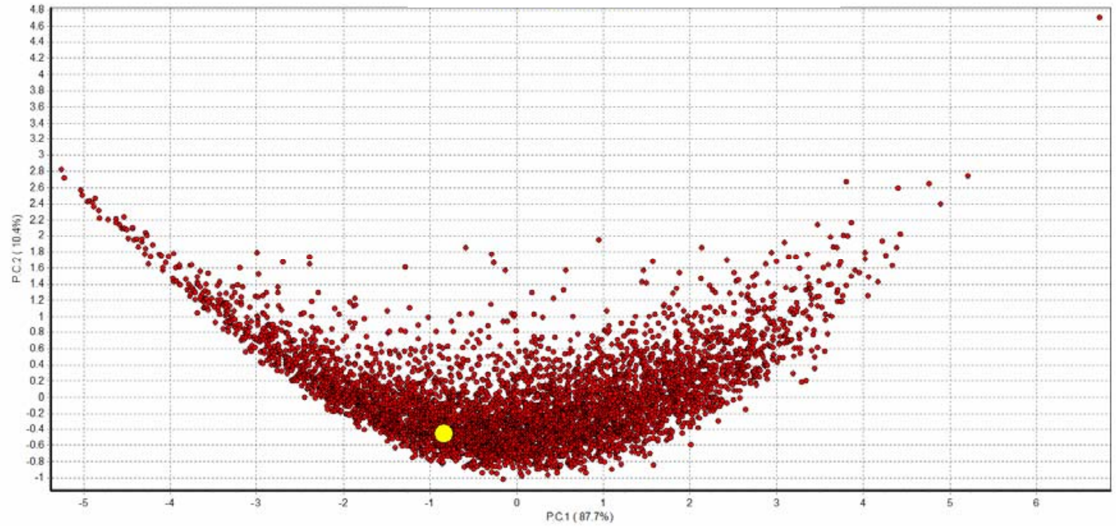

Cornuet et al BMC Bioinformatics – supplementary data file 1 – continued

(C) Probability (simulated<observed) for each of the summary statistics of the pseudo-observed test data set. Such probabilities have been computed from 10,000 data sets simulated from either the “incorrect” or “correct” prior distribution on *N*. NAL = mean number of alleles, HET = mean expected heterozygosity (Nei 1987), VAR = mean allelic size variance within population. *, **, *** = tail-area probability < 0.05, < 0.01 and < 0.001, respectively. Significant tail-area probabilities after applying the false discovery rate correction method of Benjamini and Hochberg [4] are given in red color. Note the presence of three summary statistics with very low tail area probabilities in the case of an “incorrect” prior on *N*. The high probability ($s_{\text{simulated}}<s_{\text{observed}}$) values of all three summary statistics indicate a difficulty to generate enough genetic variation within population, which fits well with the fact that the “incorrect” prior on *N* (Uniform[10; 1000]) includes only values by far lower than the “true” value of *N* = 10,000.

		Probability ($s_{\text{simulated}}<s_{\text{observed}}$)	
Summary statistics	Observed value	“Incorrect” prior on *N*	“Correct” prior on *N*
NAL	9.4000	1.0000 (***)	0.3426
HET	0.7963	1.0000 (***)	0.3349
VAR	34.5601	0.9858 (*)	0.2467

Cornuet et al BMC Bioinformatics – supplementary data file 1 – continued

Pre-evaluation of model-prior combinations: example 2. A single pseudo-observed test data set (10 microsatellite loci) was first simulated under a model of two populations (sample size of 30 diploid individuals per population) splitting at time t = 10,000 generations from an ancestral population, without subsequent migration. For all populations the effective size N = 1,000. For ABC analysis of the test data set, we used the same population and marker models, and prior distributions of demographic parameters were as followed: Uniform[100; 1000] (figure D) or Uniform[2000; 20000] (figure E) for t, and Uniform[100; 2000] for N. The mutation model and priors for microsatellite markers are the same as in example 1. We choose eight summary statistics (s): the mean number of alleles, expected heterozygosity [3] and allele size variance computed on each sample, F_{ST} values and genetic distances $(\delta\mu)^2$ between pairs of samples [5-6]. PCA on summary statistics (D and E) and probability ($s_{simulated}$<$s_{observed}$) for each of the summary statistics (F) were computed from 10,000 simulations, randomly drawing parameter values into priors.

(D) "True" value of t = 10,000 – "incorrect" prior on t (Uniform[100; 1000]): note that the observed data set (large yellow dot) is positioned outside the cloud of simulated data sets (small dots)

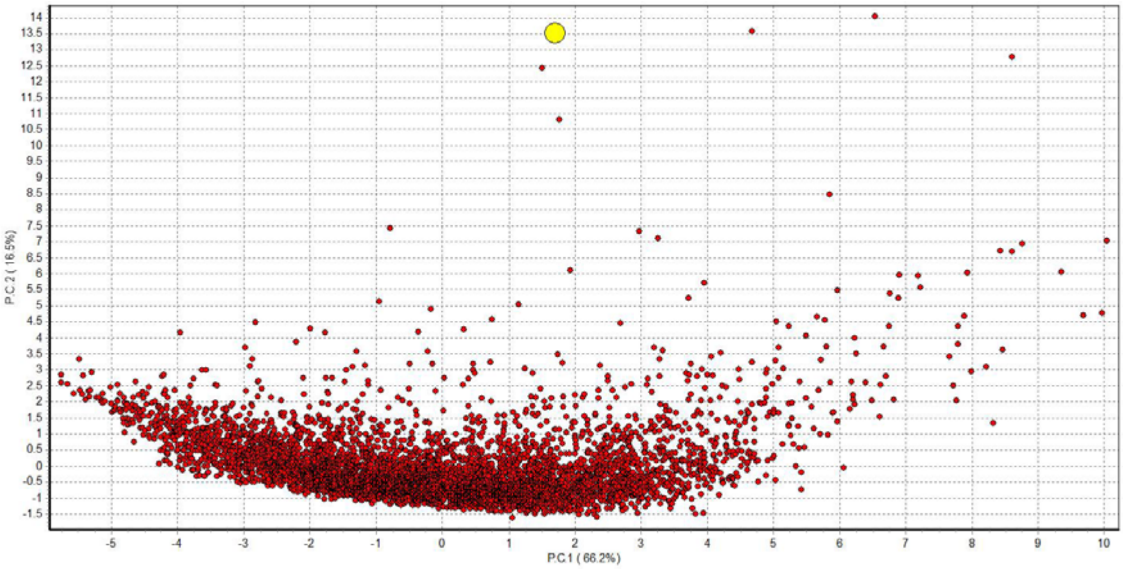

(E) "True" value of t = 10,000 – "correct" prior on t (Uniform[2000; 20000]): note that the observed data set (large yellow dot) is positioned within the cloud of simulated data sets (small dots)

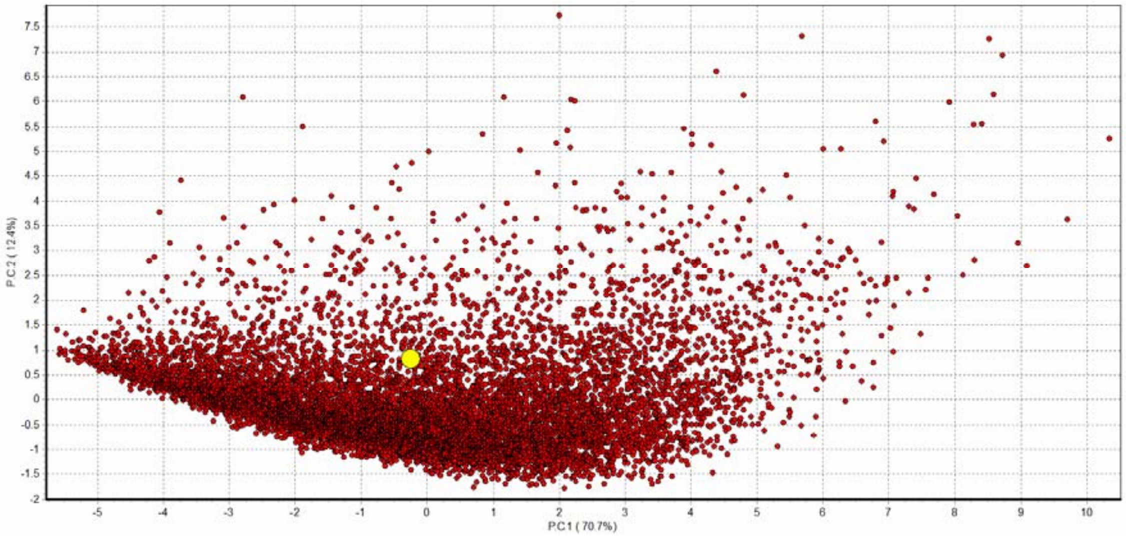

(F) Probability (simulated<observed) for each of the summary statistics of the pseudo-observed test data set. Such probabilities have been computed from 10,000 data sets simulated from either the “incorrect” or “correct” prior distribution on *t*. NAL_*i* = mean number of alleles in population *i*, HET_*i* = mean expected heterozygosity in population *i* [3], VAR_*i* = mean expected allele size variance in population *i*, FST_*i*_*j* = F_{ST} value between populations *i* and *j* [5], DM2_*i*_*j* = genetic distance $(\delta\mu)^2$ between populations *i* and *j* [6], N2P_*i*_*j*, H2P_*i*_*j*, V2P_*i*_*j* = mean expected number of alleles, heterozygosity and allele size variance pooling samples of populations *i* and *j*. *, **, *** = tail-area probability < 0.05, < 0.01 and < 0.001, respectively. Significant tail-area probabilities after applying the false discovery rate correction method of Benjamini and Hochberg [4] are given in red color. Note the presence of a summary statistics (DM2_1_2) with a very small tail area probability in the case the “incorrect” prior on *t*. This summary statistics measures genetic differentiation between populations and its high probability ($s_{simulated}<s_{observed}$) indicates a difficulty to generate sufficiently large genetic differentiation, which fits well with the fact that the “incorrect” prior on *t* (Uniform[100; 1000]) includes only values by far lower than the “true” value of *t* = 10,000.

		Probability ($s_{simulated}<s_{observed}$)	
Summary statistics	Observed value	“Incorrect” prior on *t*	“Correct” prior on *t*
NAL_1	4.0000	0.4703	0.4705
NAL_2	3.9000	0.4469	0.4493
HET_1	0.4492	0.3839	0.3811
HET_2	0.3834	0.2732	0.2731
VAR_1	13.9348	0.7442	0.7508
VAR_2	6.5267	0.4833	0.4889
FST_1_2	0.3782	0.8942	0.4378
DM2_1_2	53.7799	0.9998 (***)	0.8688

References

1. Estoup A, Jarne P, Cornuet JM: **Homoplasy and mutation model at microsatellite loci and their consequences for population genetics analysis.** *Mol Ecol* 2002, **11**:1591-1604.
2. Verdu P, et al.: **Origins and Genetic Diversity of Pygmy Hunter-Gatherers from Western Central Africa.** *Current Biology* 2009, **19**:1-7.
3. Nei M: *Molecular Evolutionary Genetics*. Columbia University Press, New York 1987.
4. Benjamini Y, Hochberg Y: **Controlling the false discovery rate: a practical and powerful approach to multiple testing.** *Journal of the Royal Statistical Society B* 1995, **57**: 289- 300.
5. Weir BS, Cockerham C: **Estimating *F*-statistics for the analysis of population structure.** *Evolution* 1984, **38:** 1358-1370.
6. Goldstein DB, Linares AR, Feldman MW, Cavalli-Sforza LL: **Genetic absolute dating based on microsatellites and the origin of modern humans.** *Proceedings of the National Academy of Sciences of the USA* 1995, **92**: 6723–6727.
