# Supplementary material for: Inference on population history and model checking using DNA sequence and microsatellite data with the software DIYABC (v1.0)
Source: BMC Bioinformatics. 2010 Jul 28;11:401. doi: 10.1186/1471-2105-11-401 (PMC2919520; doi:10.1186/1471-2105-11-401)

Cornuet et al BMC Bioinformatics – supplementary data file 2

Evaluation of the variation of RMAE values expected by chance between different replicates of 500 pseudo-observed data sets. Relative median absolute errors (RMAE) were computed for 10 replicates of 500 pseudo-observed data sets simulated under scenario 1. The data sets include 20 (independent) microsatellite loci and were generated under the scenario 1 presented in Figure 1. Parameter values were drawn from the same distributions than the prior distributions given in the legend of Figure 1. The demographic parameters *N*, *t1*, *t2*, *t3*, *t4*, *t5*, *r1* and *r2* are detailed in Figure 1. Standard deviation of RMAE values were equal to 0.009, 0.019, 0.004, 0.017, 0.012, 0.013 and 0.014 for *N*, *t1*, *t2*, *t3*, *t4*, *t5*, *r1* and *r2*, respectively. Similar levels of RMAE variation among replicates of 500 pseudo-observed data sets were obtained for other categories of genetic markers (mtDNA and nuclear sequences) and combinations of categories of markers (results not shown).

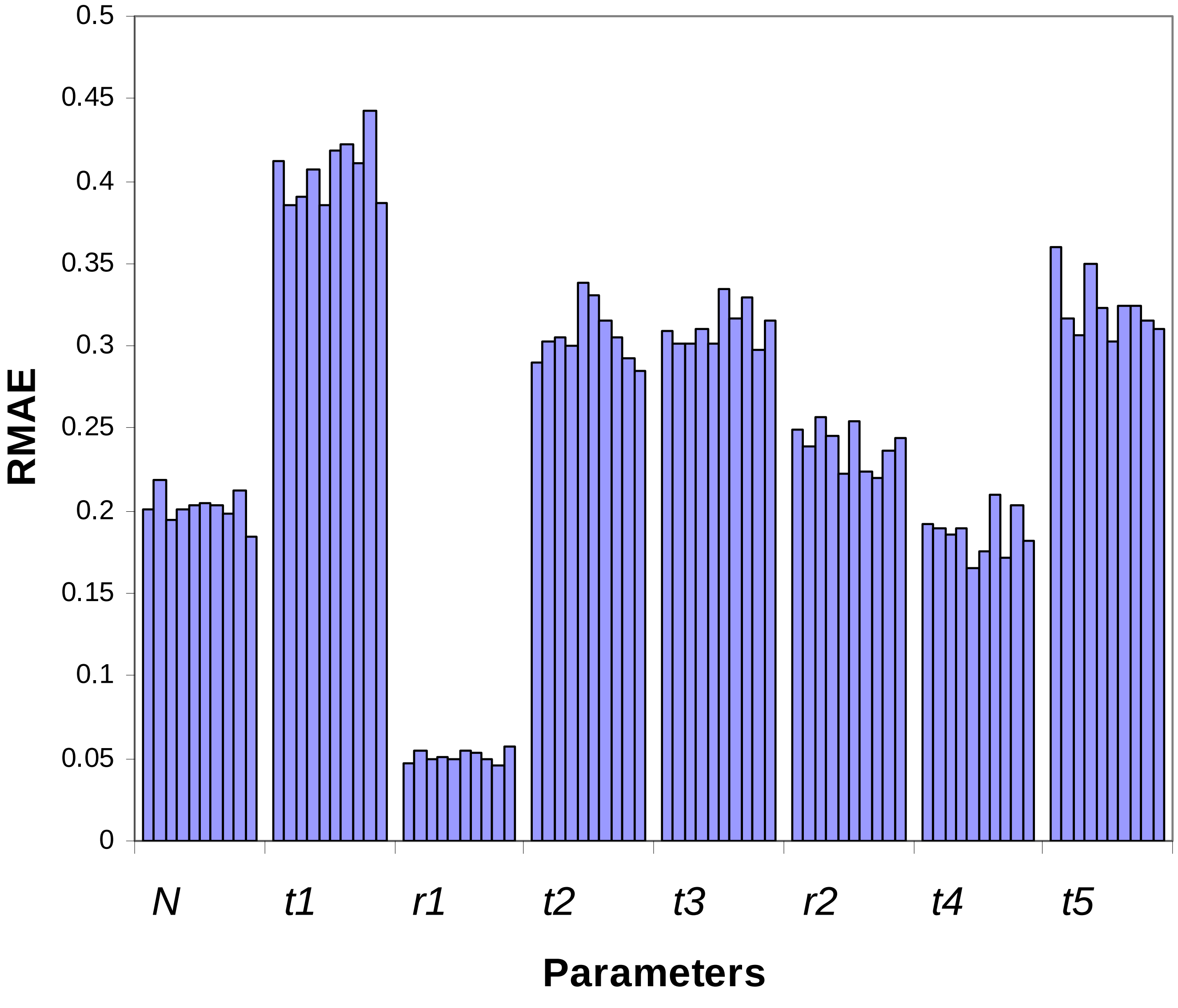

Supplement: Additional file 2 — Evaluation of the variation of RMAE values expected by chance between different replicates of 500 pseudo-observed data sets. relative median absolute errors (RMAE) were computed for 10 replicates of 500 pseudo-observed data sets simulated under scenario 1. The data sets include 20 (independent) microsatellite loci and were generated under scenario 1 presented in Figure 1. Parameter values were drawn from the same distributions than the prior distributions given in the legend of Figure 1. The demographic parameters N, t1, t2, t3, t4, t5, r1 and r2 are detailed in Figure 1. Standard deviation of RMAE values were equal to 0.009, 0.019, 0.004, 0.017, 0.012, 0.013 and 0.014 for N, t1, t2, t3, t4, t5, r1 and r2, respectively. Similar levels of RMAE variation among replicates of 500 pseudo-observed data sets were obtained for other categories of genetic markers (mtDNA and nuclear sequences) and combinations of categories of markers (results not shown). [file 1471-2105-11-401-S2.PDF]
