# Supplementary material for: Inference on population history and model checking using DNA sequence and microsatellite data with the software DIYABC (v1.0)
Source: BMC Bioinformatics. 2010 Jul 28;11:401. doi: 10.1186/1471-2105-11-401 (PMC2919520; doi:10.1186/1471-2105-11-401)

Cornuet et al BMC Bioinformatics – supplementary data file 3

The competing scenarios 1, 2 and 3 are detailed in Figure 2. The pseudo-observed test data set analyzed here was simulated under scenario 3. PCA were processed on the test quantities corresponding to the summary statistics used to compute the posterior distributions of parameters or on other statistics (see details in the legend of Table 1). Small circles = simulated from priors; large circles = simulated from posteriors.

PCA on summary statistics used to estimate parameter posterior distributions under scenario 1

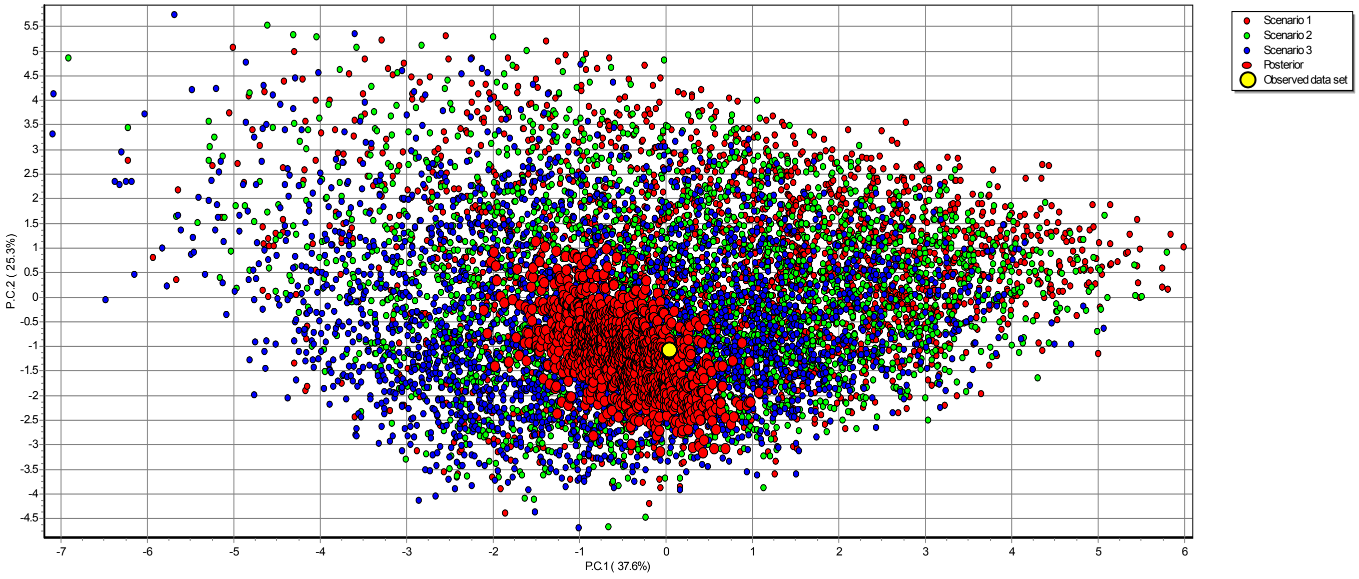

PCA on summary statistics NOT used to estimate parameter posterior distributions under scenario 1

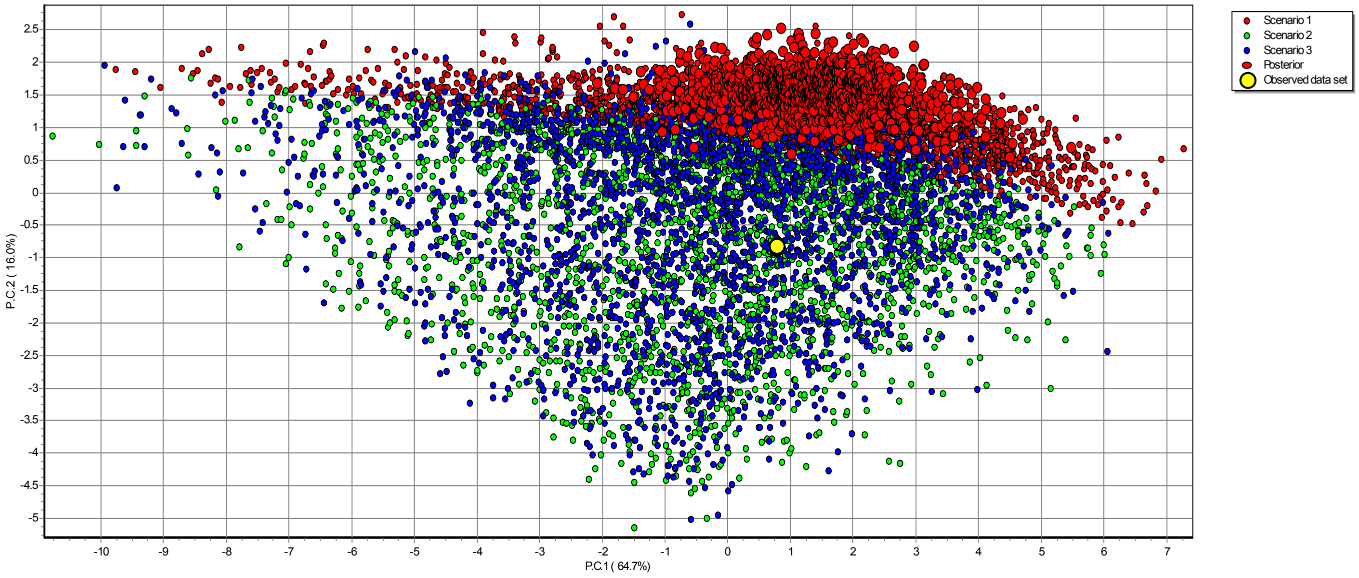

Cornuet et al BMC Bioinformatics – supplementary data file 3 - continued

PCA on summary statistics used to estimate parameter posterior distributions under scenario 2

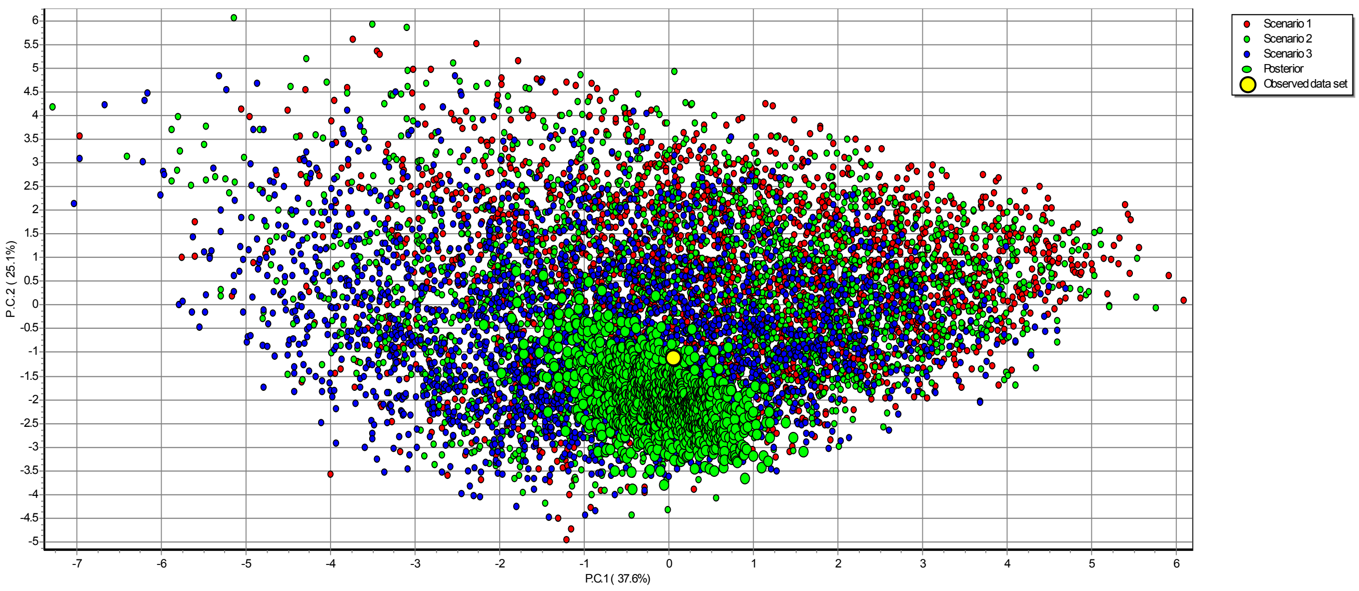

PCA on summary statistics NOT used to estimate parameter posterior distributions under scenario 2

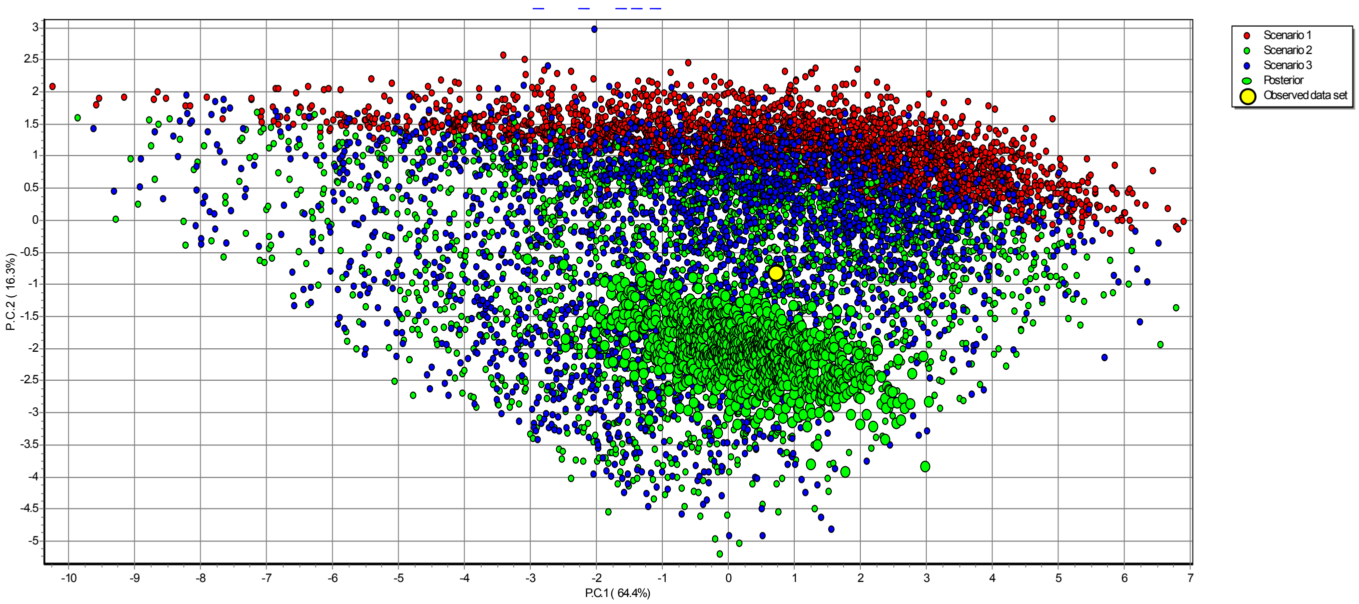

Cornuet et al BMC Bioinformatics – supplementary data file 3 - continued

PCA on summary statistics used to estimate parameter posterior distributions under scenario 3

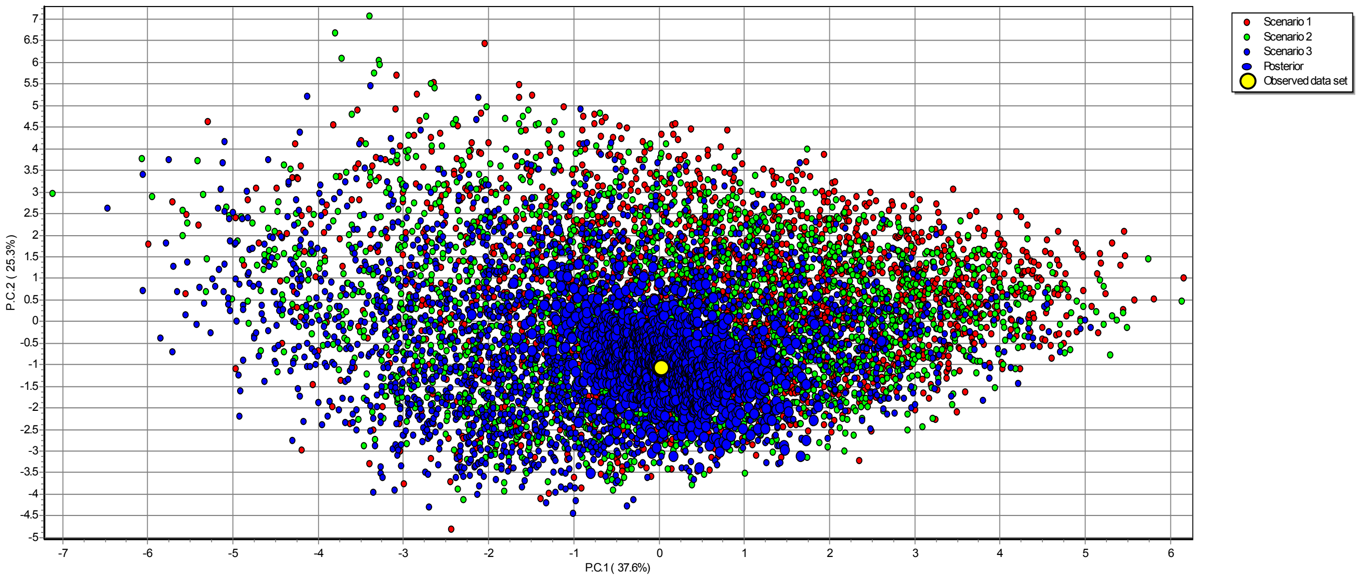

PCA on summary statistics NOT used to estimate parameter posterior distributions under scenario 3

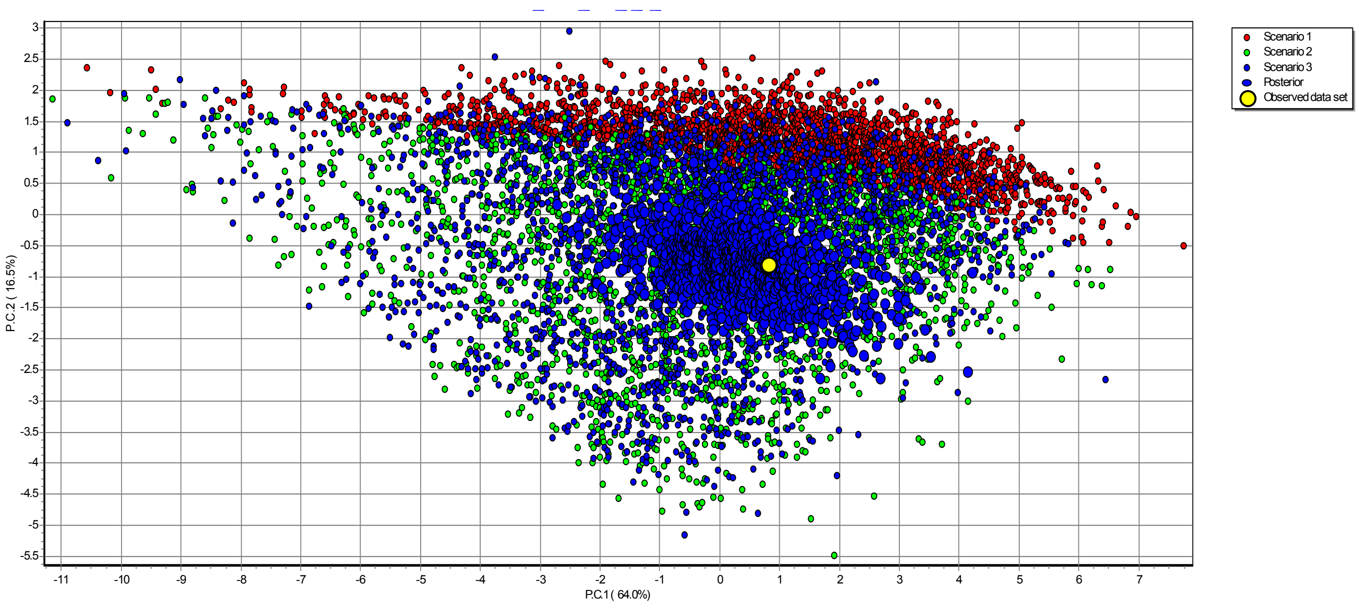

Supplement: Additional file 3 — Principal component analysis of test quantities when processing model checking for the introduction scenarios 1, 2 and 3. The scenarios 1, 2 and 3 are detailed in Figure 2. The pseudo-observed test data set analyzed here was simulated under scenario 3. PCA were processed on the test quantities corresponding to the summary statistics used to discriminate among scenarios and compute the posterior distributions of parameters (a) or on other statistics (b). The summary statistics used as test quantities are detailed in the legend of Table 1. [file 1471-2105-11-401-S3.PDF]
